# Antiviral Ranpirnase TMR-001 Inhibits Rabies Virus Release and Cell-to-Cell Infection In Vitro

**DOI:** 10.3390/v12020177

**Published:** 2020-02-05

**Authors:** Todd G. Smith, Felix R. Jackson, Clint N. Morgan, William C. Carson, Brock E. Martin, Nadia Gallardo-Romero, James A. Ellison, Lauren Greenberg, Thomas Hodge, Luis Squiquera, Jamie Sulley, Victoria A. Olson, Christina L. Hutson

**Affiliations:** 1 Poxvirus and Rabies Branch, Division of High-Consequence Pathogens and Pathology, Centers for Disease Control and Prevention, 1600 Clifton Road NE, Atlanta, GA 30329, USA; dwt4@cdc.gov (F.R.J.); kmy3@cdc.gov (C.N.M.); ioy8@cdc.gov (W.C.C.); gqu5@cdc.gov (B.E.M.); hfa5@cdc.gov (N.G.-R.); hio6@cdc.gov (J.A.E.); foe2@cdc.gov (L.G.); vao9@cdc.gov (V.A.O.); zuu6@cdc.gov (C.L.H.); 2 Tamir Biotechnology, Inc. 12625 High Bluff Drive Suite 113, San Diego, CA 92130, USA; thodge@tamirbio.com (T.H.); lsquiquera@tamirbio.com (L.S.); jsulley@tamirbio.com (J.S.)

**Keywords:** rabies virus, lyssavirus, antiviral, ranpirnase TMR-001, onconase, hamster

## Abstract

Currently, no rabies virus-specific antiviral drugs are available. Ranpirnase has strong antitumor and antiviral properties associated with its ribonuclease activity. TMR-001, a proprietary bulk drug substance solution of ranpirnase, was evaluated against rabies virus in three cell types: mouse neuroblastoma, BSR (baby hamster kidney cells), and bat primary fibroblast cells. When TMR-001 was added to cell monolayers 24 h preinfection, rabies virus release was inhibited for all cell types at three time points postinfection. TMR-001 treatment simultaneous with infection and 24 h postinfection effectively inhibited rabies virus release in the supernatant and cell-to-cell spread with 50% inhibitory concentrations of 0.2–2 nM and 20–600 nM, respectively. TMR-001 was administered at 0.1 mg/kg via intraperitoneal, intramuscular, or intravenous routes to Syrian hamsters beginning 24 h before a lethal rabies virus challenge and continuing once per day for up to 10 days. TMR-001 at this dose, formulation, and route of delivery did not prevent rabies virus transit from the periphery to the central nervous system in this model (*n* = 32). Further aspects of local controlled delivery of other active formulations or dose concentrations of TMR-001 or ribonuclease analogues should be investigated for this class of drugs as a rabies antiviral therapeutic.

## 1. Introduction

Rabies virus (RABV) is the prototype species of the genus *Lyssavirus* in the family *Rhabdoviridae* and order *Mononegavirales*. Members of this genus are the etiological agent of rabies, an acute, progressive encephalitis that affects mammals, and without proper postexposure prophylaxis results almost invariably in death. RABV is most commonly transmitted to the next host by saliva during the bite of an infected animal. Prevention of rabies through various interventions, including pre-exposure vaccination of domestic animals such as dogs and cats, and postexposure prophylaxis (PEP) in people before clinical onset are highly effective. Pre-exposure prophylaxis in humans can also be indicated in individuals at high risk, such as veterinarians, animal handlers, and laboratory personnel. For PEP, rabies immune globulin is injected around the site of the animal bite, and a regimen of rabies vaccine is administered at a distal site [1]. Despite the reduction of human rabies through canine rabies elimination programs involving elimination of stray dogs and animal vaccination programs, an estimated 59,000 people die annually of rabies from dog bites around the world [2]. In the United States, an average of two people die annually of rabies, usually transmitted by bats [3]. In some cases, aggressive treatment of the disease has been attempted in an effort to improve patient outcomes [4]. In these cases, various antiviral drugs have been used with poor results [5]. Currently no approved RABV-specific antiviral drug is available. Difficulty in delivering potential antiviral drugs to the central nervous system (CNS) contributes to the lack of effective treatment, and rabies antiviral drugs will most likely be dosed into the cerebrospinal fluid to block viral replication and spread [6].

We evaluated TMR-001, a proprietary bulk drug substance solution of ranpirnase, an RNase A enzyme, extracted from oocytes of Northern leopard frogs *Lithobates pipiens*, formerly *Rana pipiens* [7]. Other forms of the drug were developed under the names Pannon, P-30, and Onconase for Injection (TMR-004) as an antitumor agent [8,9]. Based on studies showing selective cytotoxicity in transformed cells [10], TMR-004, manufactured by Alfacell Inc., was advanced to phase III clinical trials for mesothelioma treatment [11,12]. In these trials the drug was administered to more than one thousand patients [13]. It was found to have low immunogenicity and was well-tolerated with few side effects [14]. 

Ranpirnase is currently being repurposed as an antiviral drug and is under investigation for broad spectrum activity against double-stranded DNA viruses [15], retroviruses [16,17], and single-stranded RNA viruses including Ebola virus [18]. Phase I and II clinical trials have been completed for the topical use of ranpirnase for the treatment of external genital warts caused by human papillomavirus [15]. In the case of Ebola virus, ranpirnase inhibited replication of the virus in cell culture and protected mice when administered pre- and postexposure [18]. 

The mechanism of ranpirnase antiviral activity is hypothesized as being an RNA interference pathway which can result in direct virus inhibition and alterations in the host cell gene expression [19]. The mechanism of selective cytotoxicity in transformed cells is well studied, where endocytosis [20] is followed by the degradation of dsRNA, such as tRNA, which results in halting protein synthesis and arrest of the cell cycle [21]. Proposed mechanisms of antiviral activity include degradation of viral RNA [16] and disruption of TNF-α signaling [22]. Ranpirnase is a highly stable water-soluble molecule but has never been recorded to cross the blood-brain barrier (BBB).

In the current study, we investigated the antiviral activity of TMR-001 against RABV in vitro and in vivo. This form of the drug was not previously evaluated in preclinical or human clinical studies. Transformed cell lines and a primary cell culture were used for the initial evaluation, and a transformed cell line was used to further characterize TMR-001 antirabies effect. Finally, efficacy of the compound was tested in Syrian hamsters treated and challenged peripherally.

## 2. Materials and Methods

### 2.1. Preparation of Virus, Cell Culture, and Antiviral Compound

Fixed RABV Evelyn–Rokitnicki–Abelseth (ERA) strain was propagated in BSR cells (a clone of baby hamster kidney cells) in Dulbecco’s minimal essential medium (Thermo Fisher Scientific, Waltham, MA, USA) supplemented with 10% fetal bovine serum (DMEM-10). A cell monolayer grown for three days at 37 °C and 5.0% CO_2_ was infected with a multiplicity of infection (MOI) = 1, incubated at 34 °C and 0.5% CO_2_, the medium was changed three days postinfection, and virus in the supernatant was collected seven days postinfection. Virus was titrated as described previously [23], diluted in DMEM-10 to 1 × 10^7^ focus-forming units (ffu)/mL, and stored at −80 °C. Low-passage mouse neuroblastoma cells (MNA), BSR cells, and primary fibroblast cells from *Eptesicus fuscus* (E03E) were maintained in DMEM-10 at 37 °C and 0.5% CO_2_. Cells were passaged every three or four days when the monolayer reached confluence. Cells were selected based on availability and animal models established in our laboratory (mouse, hamster, and bat). Ranpirnase TMR-001 (lot: 1602-0015) at 290 µM (3.8 mg/mL) was diluted in PBS (0.01 M, pH 7.4, without calcium, magnesium, or manganese) and stored at −80 °C. TMR-001 is the bulk drug substance used to make TMR-004 and Onconase. It contains only ranpirnase protein and mannitol. To maintain consistency, aliquots of drug and virus were only thawed and used once.

### 2.2. Cytotoxicity and Antiviral Assay

Confluent monolayers of MNA, BSR, and E03E cells were treated with 0.05% trypsin-EDTA (Thermo Fisher Scientific, Waltham, MA, USA) and diluted in DMEM-10 to 5 × 10^4^ live cells/mL using 0.2% trypan blue and counting with a Cellometer Auto1000 (Nexcelom Bioscience, Lawrence, MA, USA). A total of 5 × 10^4^ cells (1 mL) were added to each well of a 24-well plate and incubated 24 h at 37 °C and 0.5% CO_2_. The medium was removed and 10-0.024 µM of TMR-001 was added to 0.9 mL DMEM-10 in triplicate. For virus control, 0.1 mL of PBS (0.01 M, pH 7.4) was added to 0.9 mL DMEM-10 in triplicate, and plates were incubated for 24 h at 37 °C and 0.5% CO_2_. Rabies virus ERA was added to all wells at MOI = 0.1 except one untreated cell-only control well, and plates were incubated for 72 h at 37 °C and 0.5% CO_2_. Aliquots of supernatant from each well were removed at 24, 48, and 72 h postinfection and stored at −80 °C. Cytotoxicity was measured at 24, 48, and 72 h postinfection using the Neutral Red uptake assay as described previously [24]. Stored supernatant was titrated as described previously [23].

### 2.3. Time of Treatment and Cell-to-cell 50% Inhibitory Concentration (IC_50_) Assays

**Figure 1:**
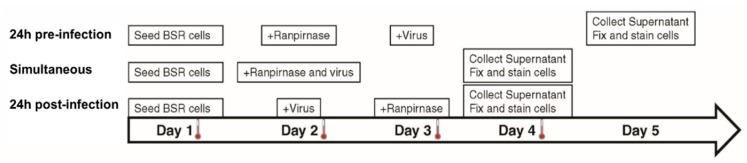
Time of treatment method. For cells treated 24 h preinfection, BSR cells (a clone of baby hamster kidney cells) were seeded in each well of an 8-well slide on day 1 and incubated overnight. On day 2, cells were treated with TMR-001 and incubated overnight. On day 3, treated cells were infected with rabies virus (RABV) strain Evelyn–Rokitnicki–Abelseth (ERA) and incubated for 48 h. On day 5, supernatant was collected, and cells were fixed and stained. For cells treated with TMR-001 simultaneously to infection, BSR cells were seeded in each well of an 8-well slide on day 1 and incubated overnight. On day 2, cells were treated with TMR-001, infected with RABV strain ERA, and incubated for 48 h. On day 4, supernatant was collected, and cells were fixed and stained. For cells treated 24 h postinfection, BSR cells were seeded in each well of an 8-well slide on day 1 and incubated overnight. On day 2, cells were infected with RABV strain ERA and incubated overnight. On day 3, infected cells were treated with TMR-001 and incubated overnight. On day 4, supernatant was collected, and cells were fixed and stained.

Confluent monolayers of BSR cells were treated with 0.05% trypsin-EDTA and diluted to 5 × 10^5^ live cells/mL as described above. A total of 5 × 10^4^ cells (0.1 mL) were added to 0.4 mL DMEM-10 in each well of an eight-well camber slide and incubated 24 h at 37 °C and 0.5% CO_2_. The medium was removed, and 29 µM–2.9 pM of TMR-001 was added in 10-fold serial dilutions to each well in duplicate for 24 h preinfection treatment and in duplicate for simultaneous treatment. Rabies virus ERA diluted in DMEM-10 was added at MOI = 0.1 to each well of simultaneous treatment slides and in duplicate for 24 h postinfection treatment (Figure 1). Drug-only, virus-only, and cell-only controls were prepared in a separate eight-well slide for each treatment. Simultaneous treatment slides were incubated for 48 h, while the 24 h preinfection and 24 h postinfection treatment slides were incubated for 24 h at 37 °C and 0.5% CO_2_. Without removing the medium, TMR-001 was added to a final concentration of 29 µM–2.9 pM (10**^−^**^1.54^–10**^−^**^8.54^ mM) in 10-fold serial dilutions to each well in 24 h postinfection treatment slides, and RABV ERA was added at MOI = 0.1 to each well in 24 h preinfection treatment slides. The 24 h preinfection treatment slides were incubated for 48 h and 24 h and postinfection treatment slides were incubated 24 h at 37 °C and 0.5% CO_2_ (Figure 1). After incubation, supernatant was collected from each well and stored at −80 °C and cells were fixed, stained, and washed as described previously [25]. Slides were observed at 200× magnification under a fluorescent microscope and clusters of greater than two adjacent infected cells (indicating cell-to-cell spread) were counted in each well and compared to counts from virus-only control wells. Stored supernatant was titrated as described previously [23].

### 2.4. Efficacy in the Syrian Hamster Model

An approved animal use protocol was established with the CDC’s Institutional Animal Care and Use Committee (protocol #2758SMIMULC-A4). Female, six-week-old, LVG Syrian hamsters were purchased from Charles River Laboratory (Wilmington, MA, USA). Animals in the intravenous (IV) treatment group underwent survival surgery at Charles River Laboratory to insert a jugular vein catheter before arriving at CDC. Hamsters without jugular vein catheters were randomly assigned to groups containing six or 12 animals (based on the design described below). We have found the hamster model to be more reproducible than mouse models, and Syrian hamsters are well-established in the literature for rabies pathogenesis studies and the evaluation of biologics. 

Animals were individually identified by implanting a microchip (Bio Medic Data System, Seaford, DE, USA) except those in IV administration groups, which were individually housed and identified by cage numbers. The microchip was preloaded in a syringe, animals were anesthetized with 1.5–5% isoflurane using a vaporizer, and the microchip was injected subcutaneously on the back. After all procedures involving anesthesia, animals were monitored continuously until fully recovered and once per day for three days after implant. 

Animals that received IV administration were anesthetized with 1.5–5% isoflurane, and starting five days after the surgery date, the catheter was flushed with 0.04 mL sterile saline and 0.04 mL lock solution (50% heparinized dextrose, SAI Infusion Technologies, Lake Villa, IL, USA) every three to five days according to the protocol provided by Charles River Laboratory. This included the day that hamsters arrived from the vendor with an IACUC waiver of the quarantine/acclimation period.

Prior to experiments with RABV challenge, 0.1 mg/kg of TMR-001 was tested without RABV challenge to ensure no neurological side effects or adverse events were observed that could interfere with the challenge study (Table S1). Group size was six animals and route of administration was different for each group: intramuscular (IM), intraperitoneal (IP), or IV every 24 h for 10 days. Animals were anesthetized with 1.5–5% isoflurane for IP and IV administration. For IV administration, the catheter was flushed with twice the capacity of sterile saline to ensure the drug entered the vein and 0.04 mL lock solution. For IM administration, animals were restrained and 0.1 mL was injected into the gluteal muscle in the left hind leg. All IM injections were done using a tuberculin syringe and needle not exceeding 28 gauge (Becton Dickinson, Franklin Lakes, NJ, USA). All groups were observed once daily for 28 days. Animals from the IP group were reused in subsequent challenge experiments after 28 days.

For the RABV challenge study, the group size was 12 female hamsters and the route of administration was different for each group as described above; an additional untreated control group was also included. Starting on day 0, 0.1 mg/kg of TMR-001 was administered by each route, as described above, every 24 h for 10 days. On day 1, all animals were anesthetized with 1.5–5% isoflurane and infected IM in the gluteal muscle of the left hind leg with 10^3.5^ mouse intracranial LD_50_ (50 µL) of canine rabies virus strain TX coyote 323R. This dose was previously determined to be lethal by the intramuscular route in the hamster model. Starting on the day of infection, animals were observed once a day, and from 7 to 21 days after infection animals were observed twice daily for clinical signs of rabies. Animals were euthanized by isoflurane overdose at the first clinical signs of rabies (e.g., paresis, paralysis, seizure), according to euthanasia criteria or at the termination of the experiment. Euthanasia was confirmed by lack of heartbeat and respiration. For hamsters not immediately undergoing necropsy, thoracotomy was used as secondary verification of euthanasia. During necropsy, the brain was harvested to confirm rabies diagnosis by the direct fluorescent antibody (DFA) test [26]. 

### 2.5. Data Analysis

The mean and standard deviation of at least four statistical replicates from at least two biological replicates were calculated. Cytotoxicity data were normalized using untreated controls, and 50% cytotoxicity concentration (CC_50_) was calculated using a linear regression. For in vitro studies, GraphPad Prism v6.07 was used to plot log_10_ transformed average and standard deviation of virus release, and to calculate statistical significance using the two-way ANOVA Dunnett’s multiple comparisons test; plot data points for virus release and cell-to-cell spread to calculate IC_50_ values using a nonlinear regression, log inhibitor vs. response, three parameter fit, and calculate statistical significance using the two-way ANOVA Tukey’s multiple comparisons test. For in vivo studies, GraphPad Prism v6.07 was used to plot average body weight and calculate statistical significance using the two-way ANOVA Tukey’s multiple comparisons test, plot Kaplan–Meyer survival curves and calculate statistical significance using the log-rank Mantel-Cox test, and analyze contingency data using the Fisher exact test. Comparisons were considered significantly different at *p* < 0.05.

## 3. Results

**Figure 2:**
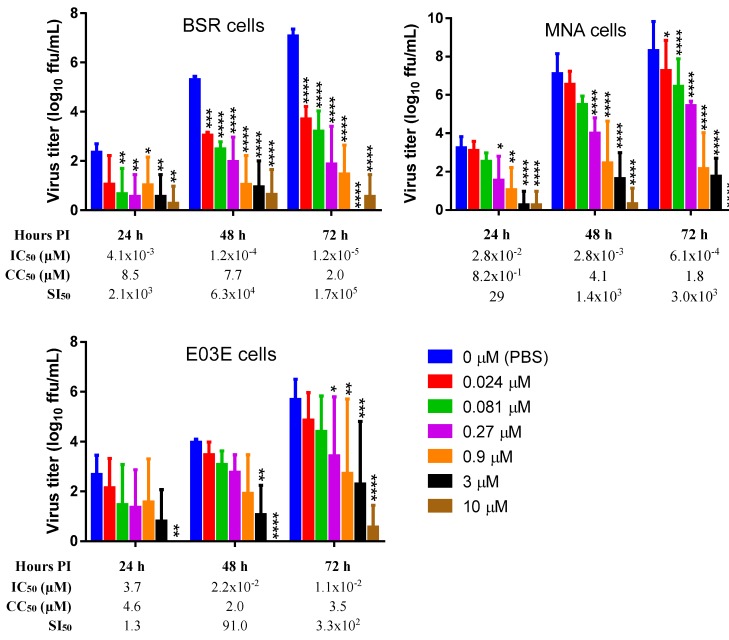
Inhibition of rabies virus release by TMR-001. TMR-001 was added 24 h preinfection at the given concentrations to a clone of baby hamster kidney cells (BSR), mouse neuroblastoma cells (MNA) and bat primary fibroblast cells (E03E). Culture supernatant was sampled at 24, 48, and 72 h postinfection. The 50% inhibitory concentration (IC50), 50% cytotoxicity concentration (CC50) and the 50% selective index (SI50) were calculated for each condition. The virus concentration in the supernatant was measured in fluorescent foci units per mL (ffu/mL) using direct fluorescent antibody (DFA) staining of MNA cells 24 h postinfection. The average and standard deviation were calculated from the log transformed data of six statistical replicates from at least two biological replicates and plotted on a linear axis. Comparison (2-way ANOVA, Dunnett’s adjusted *p*-values) of each concentration to the 0 µM TMR-001 (PBS) control are shown as <0.05 (*), <0.01 (**), <0.001 (***), <0.0001 (****). Note that no virus was detected in any of the replicates for BSR cells treated with 3 µM TMR-001 at 72 h postinfection, MNA cells treated with 10 µM TMR-001 at 72 h postinfection, as well as E03E cells treated with 10 µM TMR-001 at 24 h and 48 h postinfection.

TMR-001 was added to cell monolayers 24 h preinfection and maintained in the medium for sampling 24, 48, and 72 h postinfection. Different cell lines, due to the known cytotoxicity of ranpirnase in transformed cells [8], and a primary cell culture were evaluated. These cell lines and cell culture were selected accordingly—MNA as a standard neuronal cell model for RABV, BSR as a comparison to in vivo testing in the hamster model, and EO3E as primary cells from a common natural RABV host. The RABV titer in the supernatant of untreated cells increased approximately 2-fold at each time point (Figure 2), while virus in the supernatant decreased approximately 2-, 4-, and 10-fold with increasing drug concentration at 24, 48, and 72 h postinfection, respectively (Figure 2). The IC_50_ for ranpirnase decreased 100-fold over time (Figure 2) because the untreated virus titer increased over time and treatment inhibited viral release, i.e., the inhibition was more pronounced the longer the assay time point. The 50% cytotoxic concentration was in the micromolar range and did not change more than 5-fold over time (Figure 2). Thus, the selective index increased due to the decrease in IC_50_. The selective index was lower in the primary cell line E03E indicating that effects on the host cell may have contributed to RABV inhibition (Figure 2). 

**Figure 3:**
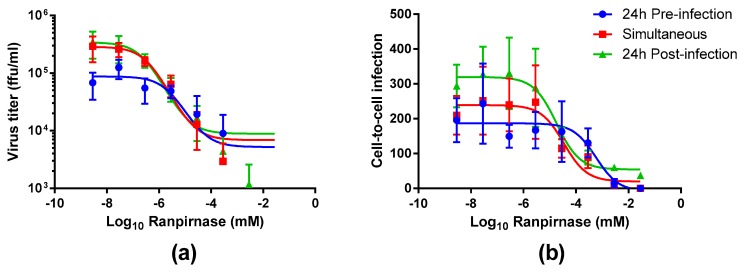
Inhibition of rabies virus release and cell-to-cell infection regardless of TMR-001 treatment time. TMR-001 was added at the given concentrations to a clone of baby hamster kidney cells (BSR) at 24 h preinfection (blue circle), simultaneously to infection (red square), or 24 h postinfection (green triangle). The average and standard deviation were calculated from four statistical replicates from at least two biological replicates, plotted on a log_10_ scale, and 50% inhibitory concentrations were calculated using a three-parameter fit, nonlinear regression. (**a**) Culture supernatant was sampled at 48 h postinfection, and virus concentration was measured in fluorescent foci units per mL (ffu/mL) using DFA staining of mouse neuroblastoma cells 24 h postinfection. Note that the titer for the first dilution (10^−1.54^ mM ranpirnase) is below the axis limit for all three treatments and for the second dilution (10^−2.54^ mM ranpirnase) is below the axis limit for 24 h preinfection and simultaneous treatments. The 50% inhibitory concentrations were 2 nM for 24 h preinfection, 0.4 nM for simultaneous, and 0.2 nM for 24 h postinfection. (**b**) The cell monolayer was fixed at 48 h postinfection, and relative cell-to-cell infection was measured by counting clusters of fluorescent foci using DFA staining and comparing counts to virus-only controls. The 50% inhibitory concentrations were 600 nM for 24 h preinfection, 100 nM for simultaneous, and 20 nM for 24 h postinfection. The amount of cell-to-cell infection was not significantly different between the different treatment time points (two-way ANOVA Tukey’s adjusted *p*-values > 0.05).

The experimental strategy was refined to better characterize the effect of different concentrations of TMR-001 on RABV infection by choosing a single time point and cell line (48 h postinfection in the BSR cell line). Additionally, the assay conditions were altered to use an 8-well chamber slide to observe the effect of ranpirnase on virus infection of the cell monolayer. TMR-001 was added to BSR cells 24 h preinfection, simultaneously to infection, and 24 h postinfection (Figure 1). The RABV titer in the supernatant increased between treatment 24 h preinfection and simultaneous treatment but was similar for simultaneous treatment and 24 h postinfection (Figure 3a). RABV titers were significantly decreased (two-way ANOVA Tukey’s adjusted *p*-values < 0.05) only for the 24 hr pretreatment group at the three lowest concentrations of ranpirnase TMR-001 (<10*^−^*^6.54^ mM). Otherwise, no other significant differences were observed, in that virus titer decreased similarly between all groups with increasing concentrations of ranpirnase TMR-001. The IC_50_ was 2 nM, 0.4 nM, and 0.2 nM for treatment 24 h preinfection, simultaneous, and 24 h postinfection, respectively.

In addition to virus released in the supernatant, the effect of ranpirnase TMR-001 treatment on RABV cell-to-cell infection in BSR cells was also measured by counting the clusters of infected cells and comparing counts to virus-only controls at each time point. This method was chosen based on an approximate 24 h replication cycle for RABV strain ERA. Using this method, all treatment groups had decreased cell-to-cell infection with increasing amounts of TMR-001, with almost no cell-to-cell infection observed at the highest concentrations (Figure 3b). Of the three treatment groups, 24 h postinfection had consistently lower cell-to cell infection at each concentration. The IC_50_ for cell-to-cell infection was 600 nM, 100 nM, and 20 nM for 24 h preinfection, simultaneous, and 24 h postinfection treatment, respectively. The amount of cell-to-cell infection was not significantly different between the different treatment time points (two-way ANOVA Tukey’s adjusted *p*-values > 0.05).

Since both RABV release into the supernatant and cell-to-cell infection were inhibited, the efficacy of ranpirnase to prevent rabies in the Syrian hamster model was assessed. First, a pilot study was completed to ensure TMR-001 treatment in uninfected animals did not cause any unexpected toxicity or clinical signs that could complicate observations of rabies clinical signs. Based on a study in mice using a different form of ranpirnase [18], a 0.1 mg/kg dose was selected with three different routes for administration: IV, IP, and IM. Overall, the animals maintained body weight while receiving ranpirnase TMR-001 (Figure S1), and gains were observed by day 28. Only the group receiving IV administration was significantly lower than for the other two groups from day 4 to day 28 (two-way ANOVA Tukey’s adjusted *p* < 0.05). No clinical signs were observed that could confound rabies clinical signs (Table S1).

**Figure 4:**
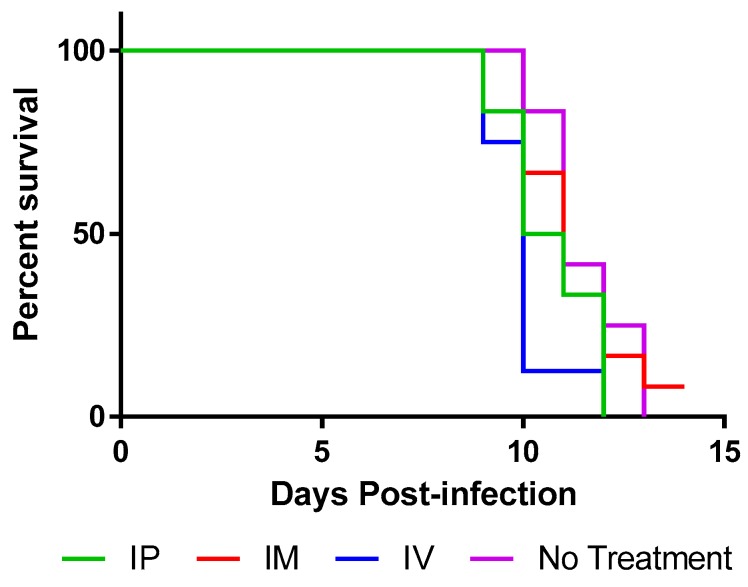
TMR-001 treatment does not prevent rabies in Syrian hamsters. Starting 24 h preinfection, 0.1 mg/kg TMR-001 was administered intraperitoneally (IP, green), intramuscularly (IM, red), intravenously (IV, blue), or no treatment (purple), once per day for 10 days. Group size was 12 hamsters, except the IV administration group, which contained 8 hamsters. All animals were infected with 10^3.5^ mouse intracranial LD_50_ of canine rabies virus IM in the hind leg. Days 7 to 21 postinfection, animals were observed twice daily and euthanized at the first clinical signs of rabies. The experiment was terminated 14 days postinfection due to a lack of significance between the groups (log-rank test *p* = 0.66). Percent survival over time is shown on a linear scale.

Next, animals were infected IM with 10^3.5^ mouse intracranial LD_50_ RABV and were treated with TMR-001 starting 24 h preinfection and continuing once per day for 10 days. The same dose (0.1 mg/kg) and routes (IV, IP, IM) were used as described above. Animals were euthanized when clinical signs of rabies were first identified, between days 9 and 13 postinfection (Figure 4) as was expected based on previous experience with the hamster model. All animals euthanized with clinical signs of rabies were positive for rabies virus antigen in the brain stem by the DFA test. One animal, from the IM group, survived to 14 days postinfection without any clinical signs of rabies but was euthanized due to lack of significant differences between the groups. This animal was tested by DFA and confirmed negative for rabies virus antigen in the brain stem at the time of euthanasia. Continuation of the experiment could not be justified because, whether the animal succumbed later or survived to the end point (45 days postinfection), the statistical significance of the groups would not change.

Survival curves for 0.1 mg/kg TMR-001 administered IP and IM were not significantly different from the control group (log-rank *p* = 0.093 and *p* = 0.66, respectively). The survival curve for 0.1 mg/kg TMR-001 administered IV was significantly lower than the control group (log-rank *p* = 0.0052) possibly due to the added stress of survival surgery to place jugular vein catheters. Median survival was similar: 10, 10.5, and 11 days for IV, IP, and IM, respectively, compared to 11 days for untreated controls. A significant number of animals 15/38 (39%) euthanized with clinical signs of rabies also showed nonspecific clinical signs (e.g., aggression, hypersensitivity, hyperactivity) prior to rabies-specific signs (e.g., paresis, paralysis, seizure) compared to previous experiments (unpublished data) in this animal model using the same challenge virus (Fisher’s exact test *p* = 0.017). In conclusion, ranpirnase TMR-001 inhibited rabies virus in vitro but not in vivo at the tested dose and routes.

## 4. Discussion

Relatively little work has previously examined the effect of ribonucleases on RABV. In 1965, Enright, et al. observed that a strain of mice resistant to rabies virus infection had higher ribonuclease activity in the peripheral blood leucocytes and serum than a susceptible strain [27]. Here, we showed that the broad-spectrum antiviral ranpirnase inhibits RABV release and cell-to-cell infection in vitro. RABV release was inhibited in a dose-dependent manner in neuronal cells (MNA), epithelial cells (BSR), and primary fibroblast cells (E03E), and cell-to-cell spread was inhibited in BSR cells. In previous studies, ranpirnase (TMR-004) protected mice at 0.1 mg/kg from an Ebola virus challenge [18] and has shown remarkable antiviral activity in vitro against other Baltimore class V viruses, including paramyxoviruses (Measles, RSV), filoviridae (Ebola virus) and, in this work, rhabdovirus.

Based on these results, TMR-001 was tested in the hamster, RABV lethal-challenge model. In the hamster model, RABV reaches the brain by 5-6 days postinfection [28]. Thus, the 10-day treatment window covers RABV entry, transit to the CNS, and replication in the brain. Results of TMR-001 administered at 0.1 mg/kg by three different routes were not significantly different for clinical onset or death within this animal model, demonstrating additional research is warranted to evaluate RNases as a potential rabies antiviral. That one of the animals treated by the IM route survived 14 days postinfection without any clinical signs of rabies is promising and may indicate a protective effect. Indeed, only one other RNase (from *Bacillus intermedius*) has been tested in animals for rabies antiviral activity, and in that case Gribencha, et al. found only injection of 5 mg/kg at the site of infection was prophylactic [29]. If this class of drugs is found to be noninferior and more cost-effective than rabies immune globulin, then it could be considered as a replacement for rabies immune globulin in postexposure prophylaxis.

While ranpirnase has an antiviral effect in vitro, effectiveness of this compound in vivo likely requires the ability to cross the BBB or novel delivery methods. A form of ranpirnase (Onconase for Injection) was rapidly distributed throughout the body and had a short serum half-life of 36.5 min [30]. However, the drug appears to remain active in the cell for up to 4 days [16]. In another study, a recombinant ranpirnase peptide was targeted to neurons when injected directly into the cerebrospinal fluid [31]. Further studies of ranpirnase activity in the CNS will be informative to understand the potential of TMR-001 as an antirabies therapy.

This study further supports the need to understand the mechanism of action of potential antiviral compounds when the infections cross the BBB. RABV-specific antiviral compounds, which are viricidal, will most likely have to pass through the BBB to be effective, particularly for IV or oral routes of administration [6]. Alternatively, this effect may be overcome by direct administration into the cerebrospinal fluid. In a mouse model, administration of mannitol was shown to increase the permeability of the BBB but had no effect on RABV challenge [32]. Changes could also be introduced into potential drugs such as acylation that could potentially improve transport through the BBB [33], or RNases could be linked to other proteins or antibodies to better target RABV infection [34]. Future studies could incorporate changes to TMR-001, increased concentrations, and increased permeability of the BBB to potentially improve clinical outcomes. Further studies could evaluate the effects of RNases to prevent rabies in other animal models as this study was confined to the Syrian hamster model.

Recently interest in favipiravir as an RABV antiviral [35] has increased after reports of human rabies survivors treated with this drug [36]. Additional, studies of favipiravir showed in vitro inhibition of RABV but no effect on rabies survival in a mouse model [37,38,39]. Recently, in vivo imaging was used to show favipiravir suppressed RABV replication in the periphery, but double or triple the dose was required to suppress replication in the CNS [40]. Another recent study also showed in vitro efficacy but limited effect in vivo of antimicrobial peptides dermaseptins against RABV in a mouse model [41]. These studies demonstrate that the in vivo delivery of rabies antivirals in animal models is a confounding factor that must be addressed in future rabies antiviral studies.

In conclusion, ranpirnase is a promising broad-spectrum antiviral that inhibits RABV in vitro. Additional studies are required to determine if an effective dose, route of delivery, new formulations of the drug, or another RNase A homologue could prevent rabies in an animal model. The need for an effective RABV antiviral drug is of utmost importance due to the high lethality of natural infection and dearth of medical countermeasures once symptoms manifest.

## Supplementary Materials

The following are available online at https://www.mdpi.com/1999-4915/12/2/177/s1, Figure S1: Effect of TMR-001 administration on body weight in healthy animals, Table S1 Observation of Syrian hamsters administered TMR-001 once/day for 10 days.
