# Supplementary material for: Antiviral Ranpirnase TMR-001 Inhibits Rabies Virus Release and Cell-to-Cell Infection In Vitro
Source: Viruses. 2020 Feb 5;12(2):177. doi: 10.3390/v12020177 (PMC7077210; doi:10.3390/v12020177)

Table S1. Observation of Syrian hamsters administered TMR-001 once/day for 10 days

Group	Appearance	Paresis	Paralysis	Seizure	Self-mutilation	Notes
0.1 mg/kg IP	100% (6/6) redness on lateral ventral side by day 7	0% (0/6)	0% (0/6)	0% (0/6)	0% (0/6)	Mild skin reaction
0.1 mg/kg IM	Normal	0% (0/6)	0% (0/6)	0% (0/6)	0% (0/6)	
0.1 mg/kg IV	Normal	0% (0/5)	0% (0/5)	0% (0/5)	0% (0/5)	1 euthanized due to problem with catheter day 0

Figure S1. Effect of TMR-001 administration on body weight in healthy animals

TMR-001 was administered intraperitoneal (IP, green), intramuscular (IM, red), or intravenous (IV, blue) at 0.1 mg/kg once-per-day for 10 days, and animals were observed for 28 days. Group size was 6 hamsters; except IV administration group size was 5 hamsters. The mean body weight in grams (g) and standard deviation for each group over time is shown. By day 4 and continuing to day 28, the average body weight for the IV administration group was significantly lower than both IM and IP groups (two-way ANOVA Tukey's adjusted *p*-values <0.05). No other differences were significant. No clinical signs were observed in any of the groups.

Fig. S1

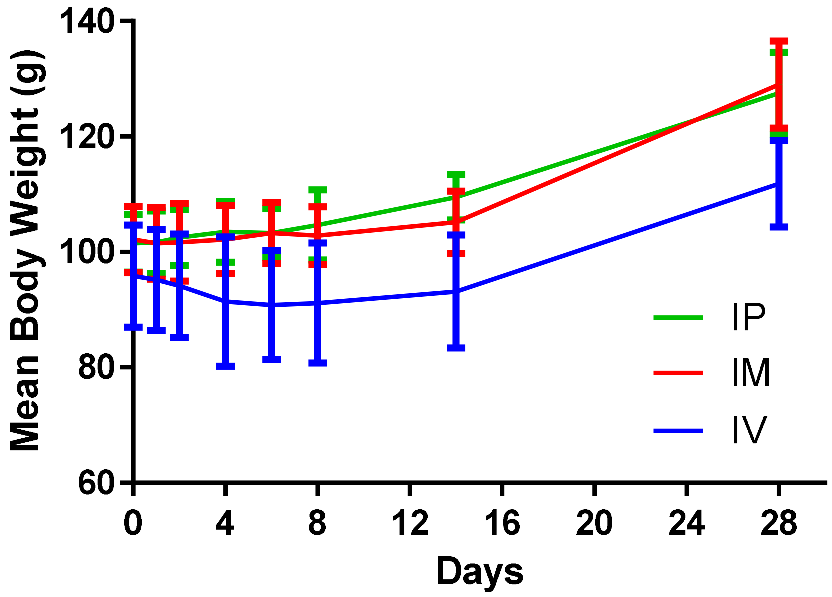

Supplement: Supplementary file 1 [file viruses-12-00177-s001.zip › viruses-657082-supplementary.pdf]
